# Complex System Approaches for Animal Health Surveillance

**DOI:** 10.3389/fvets.2019.00153

**Published:** 2019-05-16

**Authors:** John Berezowski, Simon R. Rüegg, Céline Faverjon

**Affiliations:** ^1^Vetsuisse Faculty, Veterinary Public Health Institute, University of Bern, Bern, Switzerland; ^2^Vetsuisse Faculty, University of Zurich, Zurich, Switzerland

**Keywords:** animal health surveillance, animal disease surveillance, complex adaptive system, complex systems, systems science, food animal production, food animal systems, food animal value chains

## Abstract

Many new and highly variable data are currently being produced by the many participants in farmed animal productions systems. These data hold the promise of new information with potential value for animal health surveillance. The current analytical paradigm for dealing with these new data is to implement syndromic surveillance systems, which focus mainly on univariate event detection methods applied to individual time series, with the goal of identifying epidemics in the population. This approach is relatively limited in the scope and not well-suited for extracting much of the additional information that is contained within these data. These approaches have value and should not be abandoned. However, an additional, new analytical paradigm will be needed if surveillance and disease control agencies wish to extract additional information from these data. We propose a more holistic analytical approach borrowed from complex system science that considers animal disease to be a product of the complex interactions between the many individuals, organizations and other factors that are involved in, or influence food production systems. We will discuss the characteristics of farmed animal food production systems that make them complex adaptive systems and propose practical applications of methods borrowed from complex system science to help animal health surveillance practitioners extract additional information from these new data.

## Introduction

Producing food from farmed animals is a large and very complex process that occurs in integrated networks made up of many individual animal producers, supporting businesses (veterinarians, feed suppliers, animal haulers, animal marketers etc.), and processors (slaughter plants) (1–4). Farmed animal food has many names including Agri-Food systems, food animal value chains, food supply chains, livestock production systems, and others. For simplicity we will call them Food Animal Systems (FAS).

Many of the individuals and businesses participating in FAS currently collect and store large quantities of data. The purpose of these data are to manage the many individual activities that contribute to food production, and these data are often stored in different databases that are controlled by many different individuals and organizations participating in the FAS. The list of individual data types is long and includes, but is not limited to: farms (unique farm ID, location, production types, production capacities, population sizes, production practices, nutrition, biosecurity etc.), production performance (feed consumption, milk production, weight gain etc.), reproductive performance (conception rates, days to estrus, birth rates etc.), disease prevention (vaccinations, anti-parasite treatments, preventative antimicrobial treatments, etc.), clinical disease (farmer reported disease symptoms, abortions, deaths etc., veterinarian reported clinical signs, postmortem findings, field diagnoses, herd level morbidity, etc., diagnostic pathology laboratory data including patho-anatomical and etiological diagnoses, diagnostic test results etc.), disease treatments (antimicrobial and other pharmaceutical prescription data, etc.), animal movements (date, time, number and production type of animals moved, origin and destination, identity of the truck moving the animals, etc.), animal carcasses (date, time of slaughter, carcass characteristics, lesions and other reasons for partial or complete carcass condemnation, etc.), and others (5–10). These data are produced continuously, many are geo-located, and many are time stamped allowing them to be analyzed as time series. These data are also highly variable because they are entered by many different people into many different software platforms, with no common data entry standards. Because of the large volume, high rate of production and large variability of these data, they could be called Big Data. If lessons are to be learned from Big Data analyses in other fields, these data, if combined and analyzed together, hold an opportunity for creating new information (11).

Animal health surveillance organizations are beginning to centrally collect and analyze some of these data to enhance animal health surveillance (AHS) (5). Analyzing large volumes of highly variable data is a challenge for AHS practitioners, as the methods currently used in AHS are not adapted to large quantities of highly variable data. AHS organizations tend to be relatively narrow in their scope of activities, often focusing surveillance activities (specifically data collection and analyses) on creating information needed to deal with single disease issues (12). A similar approach is being used to deal with these new data (5). Current analytical approaches for dealing with these new data focus primarily on syndromic surveillance(SS), a method which aims to detect disease epidemics by identifying unusual events in time series (5, 6). Most SS used in AHS are univariate in that they monitor single time series (5). Multivariate monitoring methods are being explored for dealing with multiple time series (13, 14). However, these approaches are also relatively narrow in scope as they focus mainly on identifying unusual events in the data, and ignore much of the other information that could be extracted from these data.

We suggest that a new analytical paradigm should be adopted for dealing with these new and highly variable data. Since animal derived food is produced in large complex systems, a broader, more holistic analytical approach could produce new information with additional benefits not only for early epidemic detection, but also for producing evidence of disease freedom and enhancing our understanding of the processes leading to disease occurrence. The complexity of FAS makes them analytically challenging, however systems with similar characteristics are very common and researchers have developed analytical and modeling methods adapted to these systems.

Complex System Science (CSS) is a broad term used to refer to the study of complex natural and manmade systems, such as animal-based food production systems. Other terms used for studying complex systems include Complexity Science and System Science.

In public health, CSS methods have been used to gain a greater understanding of the systems that influence population health with the aim of improving decision making in health policy by identifying intervention points having positive health outcomes with minimal unintended consequences (15). Recent examples where CSS have been applied in public health settings include: a framework to understand, communicate and develop action strategies for cardiovascular disease and diabetes (16), understanding infectious disease epidemic dynamics in order to make policy decisions relating to vaccination programs, epidemic responses and other infectious disease control programs (17), understanding nursing as a complex adaptive system (18), gaining a better understanding of, and promotion of oral health equity (19), understanding determinants of inequities in healthy eating (20), and improving alcohol misuse policy (21). For reviews, see Rusoja et al. (22), Chughtai and Blanchet (23), Carey et al. (15), and Luke and Stamatakis (24).

In veterinary public health (VPH), CSS methods have been applied to FAS in order to better understand the effects of disease and other factors on these systems with the goal of developing more effective disease control, food security and food safety policies with minimal unintended consequences (25). Examples from VPH include: modeling of the beef supply chain in Botswana to estimate the effects of various policies on different actors in the supply chain (26), modeling the small holder pig producer network in Vietnam to evaluate the impact of animal health and food safety issues on different actors in the supply chain (25), modeling the dairy supply chain in Nicaragua to estimate the multidimensional impacts of policies and interventions on value chain actors (27), modeling the beef supply chain in Zambia to estimate the influence of socioeconomic, cultural and economic factors on East Coast Fever in order to identify targets for more effective disease control policy (28), and modeling of small holder pig value chains in Uganda to conduct an ex-ante assessment of the impact on value chain actors of biosecurity interventions aimed at controlling African Swine Fever (29). For a review of CSS modeling in veterinary public health, see Rich et al. (25) and Lanzas and Chen (30).

In spite of the aforementioned benefits of CSS in public health and veterinary public health, and publications calling for the use of CSS in public health (31) and animal health surveillance and disease control (25, 32), to the best of our knowledge these methods have not been used to deal with the complex FAS data that AHS practitioners are currently struggling with. In this manuscript, we will briefly introduce complex adaptive systems (CAS) and CSS, discuss characteristics of FAS that make them CAS, and provide examples of how CSS methods could be used to help deal with the large data volume becoming available in AHS.

## Complex Adaptive Systems (CAS)

Complex Adaptive Systems are ubiquitous in our world (33, 34). The term has been applied to many natural (cells, animals, plants, ecosystems, weather) and man-made (cities, economies, factories) systems (35). The characteristics of CAS make them by nature not simple, easy to understand, or even easy to study (36). There is no concise definition of a CAS (36). For the purposes of this manuscript we will define a CAS in simple terms, as a loosely bounded group of different types of independent dynamic agents (also called entities, components, agents, elements, units, and other terms) and the interactions they have with each other and the environment in which they are located. There can be many different types of agents within a single CAS. Agents vary greatly in size and complexity; some are tangible (material) and some are intangible such as market prices, laws, beliefs and cultural habits. They can be small, for example an atom or molecule, or large, for example a large weather system such as a hurricane, and can include almost everything in between the two extremes. Many of the agents within a CAS are themselves CAS. There can be many different types of interactions between agents within a single CAS, including many different types of predation, parasitism, commensalism, cooperation, communication and others. Adaptive refers to the dynamic nature of CAS. They are not stationary; they change constantly because of the interactions between entities and the interactions between entities and the environment. In addition new often unpredicted properties, such as new organizational structures or new dynamics may emerge. These characteristics have important consequences for studying CAS. Research approaches that estimate causal associations by capturing empirical data on a few potential causal factors at one point in time may not sufficiently estimate the dynamic interactions that occur between the many agents in a CAS. For a general description of the properties of CAS, see Kwapien and Drozdz (37) and Bar-Yam (34).

## Properties of Food Animal Systems that make them Complex Adaptive Systems

Food animal systems have many properties that characterize them as CAS (1, 38). We will consider a small list, including only characteristics that are relevant to the purpose of this discussion, as a complete description is beyond the scope of this manuscript.

The list of independent agents in FAS is long and includes, but is not limited to: animals, farms, farmers, animal product processors (meat, milk, egg, and other products), animal and animal product transporters, traders, feed suppliers and the microbial organisms (viruses, bacteria, protozoa etc.) that inhabit animals, people and their environments, market prices, legal frameworks, production standards, social conventions, traditions and many more. These agents are organized into hierarchies with the highest level being the whole FAS. The overall system is composed of individual interconnected production systems made up of individual farms, feed supply companies, veterinarians, and animal transport companies. Individual farms are made up of farmed and non-farmed species (livestock, pets, rodents, wild animals, insects, plants, pathogenic and non-pathogenic microbes), farmers and their families, and the environment in which they are situated. Animals are made up of smaller subunits (organs and organ systems), which in turn are made of smaller subunits (individual cells), and these are composed of yet smaller sub-units (cell organelles and metabolic pathways) and so on.

Within FAS there are many different interactions constantly occurring between different agents, between agents and the environment, and between agents and other external organizations (for example legislation or the influence of international markets on local markets). Examples include interactions within individual farms (between animals, people, microbial organisms, and the environment) and between farms (movements of animals, people, feed, veterinarians and others between farms). Interactions can result in feedback loops within the FAS. For example, in free market economies, increased production can negatively affect the market price of animals, which in turn can modify farmer behavior to decrease production, which in turn can affect the market price and so on. Some of these interactions have been well-described and extensively modeled for understanding the transmission of disease in populations of animals and across networks of farms (30, 39, 40).

One of the most important characteristics of FAS, and CAS in general is their dynamic nature. The flow of animals, animal products, other materials and information though FAS combined with the many interactions occurring in FAS means that FAS are constantly changing. The dynamic nature of FAS has consequences for designing epidemiological studies of these systems. For example, the results of cross sectional studies of FAS may quickly become invalid because the FAS may change significantly after the study has been completed. Much of the data collected from FAS is collected on a continuous basis and can be converted into time series. Study designs and analytical methods used to study FAS should be adapted to time series and the analyses should updated on a regular, frequent basis.

Complex adaptive systems do not have clearly defined, stationary boundaries. Rather they are open in the sense that CAS do not function independent of outside influences, rather they receive outside inputs (material, information, and other types) at different hierarchical levels of the system. Weather for example can have a direct effect at the animal level (for example cold wet weather is associated with an increased risk of neonatal diarrhea in newborn calves). It can also have indirect effects. Dry growing conditions can drastically reduce feed crop yields resulting in increased prices for certain feeds, making them uneconomical for farmers to purchase, forcing farmers to search for alternative often lower quality feeds that can affect farmed animal nutrition across the complete production system. Other external inputs include legislation aimed at regulating farmed animal production and market prices for animals and animal products that can influence the resources that farmers have available for nutritional and other management inputs that can affect animal health and production. The indeterminate and constantly changing nature of the boundaries of CAS create some challenges for studying them, especially for developing CAS models. Setting limits to a FAS boundary in order to model a FAS is an arbitrary process, and where the limits are set will affect our understanding of the CAS. For example if disease control legislation is included in a model or not will have an effect on the performance of the model and any understanding of the behavior of the FAS derived from studying the model.

There is legislation in many jurisdictions aimed at regulating or influencing some of the behaviors of FAS. However, FAS are not planned, rather they are self-organized. The characteristics and current form of a FAS is the result of a large number of external and internal factors that have favored the initial formation of the system and then influenced it over time. Depending on the FAS, these could include the characteristics and availability of agricultural land, availability of other needed resources (for example water, fertilizer, seed for forage and feed crops), favorable climatic conditions, livestock species adapted to the region and production systems, adequate human resources, markets for the products of the system, commodity prices that can support profitable production, and the list can go on.

Finally, these systems have emergent properties that are difficult to explain by understanding the properties of the individual agents in the system. In other words, a reductionist approach where a system is broken into individual component parts (animals, farms, pathogens) that are individually studied may not provide sufficient information to understand and predict the behavior or properties of the system as a whole. This is because a reductionist approach is limited in that it largely ignores the interactions occurring between components in the system (38). Disease epidemics have been reported to be an emergent property of FAS, and the problem of preventing them, understanding the conditions that promote them and predicting them is considered a CSS problem (30). This is strong motivation for broadening the scope of AHS activities beyond a single disease focus. A more holistic approach that considers disease to be a product of the FAS as whole may be a more productive approach, resulting in the creation of surveillance information that has more value for disease control (32).

Considering the large number of participants and the complex nature of FAS, it should not be surprising that once computer technologies became widely adopted within FAS that large volumes of highly variable and complicated data would be created by FAS participants. It should also be no surprise that analytical approaches for understanding FAS and extracting information from these complex data will require specialized analytical approaches and methods fit for the task.

## Challenge for Animal Health Surveillance

The purpose of AHS is to produce information to aid in decision making in animal disease control (41). In general, the target of AHS information producing activities is relatively narrow, limited to diseased animals, non-specific data produced by diseased animals, or metrics of human behavior changes in response to diseased animals (12). The most common activities used in AHS focus on finding and counting diseased animals, or proving the absence of diseased animals in a population (41).

Risk-based surveillance focuses surveillance activities on specific subpopulations or geographical regions with the highest risk of disease occurrence. It is a somewhat broader approach, as it incorporates a variety of information about different factors associated with disease occurrence (42). However, the methods used for risk-based surveillance are not adapted to time series analyses and therefore are not suitable for understanding the dynamic nature of FAS.

Syndromic surveillance focuses mainly on monitoring time series of metrics (milk production data, abortions, deaths, veterinary visits to farms, lesions seen at slaughter in previously diseased animals and many others) associated with disease occurrence in animals. The purpose of SS is to identify changes (or signals) in these time series such as statistically unusual or extreme increases or decreases in time series values that could be early indicators of recently started epidemics (6). Currently, SS approaches used in AHS are mostly univariate in nature, focusing on identifying abnormal signals in single times series. Multi-variable approaches aimed at identifying abnormal signals in multiple time series are being explored, but are not widely implemented at this time (5). Multi-variable SS uses more of the data produced by FAS, however, the information produced is limited only to identifying signals in the data that could be produced by disease epidemics. Furthermore, multi-variable SS is not being used to understand the complex, dynamic nature of FAS or how FAS as a whole produce disease.

Epidemiologists who are well aware of the complex multifactorial nature of disease causality in populations have not widely adopted CSS in their work. Epidemiological causality studies are generally interested in reducing large numbers of potential causal factors to a small number of the most important, proximal-in-time determinants of disease (43). Even though some of these studies aim to identify causal associations that exist over time (for example in longitudinal studies), methods aimed at understanding the dynamic nature of the interactions between causal and other factors in FAS and how these interactions result in disease occurrence are not widely used by epidemiologists (43).

The challenge for AHS practitioners is to view these new data as a new opportunity. These data have the potential to provide a more complete understanding of the processes that result in disease production, which is of central importance for disease surveillance. The large volume of data that is being produced by FAS has not been previously available, and it is understandable that AHS practitioners have not yet built a tool kit fit for dealing with these data. It is clear that if the epidemiologists working in AHS are going to exploit these data to improve surveillance, they will need to enhance their current analytical tool kit, and this will requires looking beyond the current approaches used in surveillance. In this manuscript, we will limit the discussion to CSS methods with potential utility in AHS including: (1) models that are used to better understand disease and the data produced from FAS and (2) methods for identifying impending or ongoing change in the behavior of a CAS that could be caused by or associated with an increase in disease in a FAS.

## Modeling Food Animal Systems using Complex System Science Methods

The ubiquity of CAS has attracted many researchers from different disciplines who have applied their own approaches to understanding CAS (18, 44). Out of these efforts, CSS has emerged as the broad interdisciplinary field that aims to study, describe and understand CAS (24). One of the interesting findings of CSS is that even though there are a seemingly unending variety of CAS in the world, many or all of them share some basic characteristics (18). One of the goals of CSS is to discover basic principles or laws that underlie the formation and behavior of CAS (45). From a practical point of view, these commonalities suggest that the methods and approaches that have been successful in studying one type of CAS may have potential application for studying others.

In recent years modeling methods have become the most commonly used approaches for studying CAS. Reasons for this are the availability of computer technologies to support their use and the need for new tools specifically designed to deal with the challenges of CAS (30, 39). The overall purpose of creating and using dynamic models of CAS is to gain a better understanding of how the CAS being modeled functions. In veterinary public health these models are often used to evaluate the costs and benefits of surveillance, food safety measures, disease control programs, and other interventions (25, 28, 30). We suggest using similar processes for building models of FAS, but with the purpose of strategically and practically dealing with data from the FAS.

Several modeling platforms have used in public and animal health including Social Network Models, Agent Based Models, and Systems Dynamics Models (30, 39, 46).

Social network models, and social network analysis focus on the interactions (animal movements, contacts between individuals, animal or animal product trade) between participants within network (47). They have been used as stand-alone applications or in combination with System Dynamics Models(SDM) to identify individuals within networks that have a high risk of propagating epidemics, and predicting the potential size of epidemics in order to optimize surveillance and response to disease introductions (47).

Agent based models simulate the behavior of individual agents and the interactions between agents based on user defined properties for each agent and for the system as a whole. They have several advantages including their ability to:(1) easily model heterogeneity within and between individuals, (2) model spatial (geographic location) characteristics, including stochasticity, (3) they are dynamic, allowing simulation of model progress over time which is important for exploring the potential effects of changing parameters on the model performance (30), and (4) they can accommodate other models such as SDM (48). Agent Based Models have become favored platforms for modeling large scale epidemics (30) and have been used to model agri-food supply chains for understanding the behavior of farmers (49), and farmer decision making (50).

System Dynamics models are used to model the relationships between different participants in a CAS by modeling flows, such as the flow of animals, products, information, pathogens and other things through the FAS. They can simulate the effect over time of various internal or external changes on individual CAS participants and the CAS as a whole (25). For example they can model the effect of farmer behavioral changes on disease production, the effect of new regulations, or the effect of market prices changes on participants in the FAS and the behavior of the FAS as a whole (51). They have also been used to model the dynamics of disease epidemics (52, 53) and the effect of production practices and other risk factors on disease dynamics in animal production systems (54). Quantitative SDM use computer simulation to model the dynamic changes that occur in CAS over time. In animal health quantitative SDM (also called Susceptible, Infected Recovered or SIR models) have been used to estimate the effect of various polices, such as disease control, food safety or supply management on participants in food animal value chains (25). System Dynamics Models can be qualitative, such as causal loop diagrams (55), focusing on modeling the structure of the CAS by identifying the important participants in the CAS and their relationships. In food animal value chains they have been used in a participatory fashion where stakeholders are engaged in defining the model structure, identifying important value chain participants and the relationships between them (28, 51, 55).

Each of the modeling platforms has advantages and disadvantages, and one modeling platform alone may not be sufficient for a national FAS. Fortunately, it is possible to combine platforms, and it may be that using multiple modeling platforms together will be the most useful approach.

A participatory model building process will be critically important. The benefits of including a range of stakeholders in the FAS modeling processes (27, 28, 51) and animal health surveillance (56, 57) have been reported. In our opinion including a wide range of stakeholders representing many or all types of FAS participant groups will be especially important for developing a valid and useful FAS model. Surveillance practitioners dealing with these data are, in most cases, external from the FAS, and do not have regular frequent communication with participants in FAS. In general, they will not likely have adequate knowledge to create a useful dynamic model of a FAS. They will not have knowledge about which FAS participants are most important to include in the model, or the perspective of many of the participants in the FAS, particularly in terms of their understanding of the accuracy and value of the data they create and submit to the surveillance system. Stakeholders from the FAS can provide this knowledge as well as information about participant characteristics (for example production practices) and the characteristics of interactions between participants (for example communication) that may not be evident in the data they provide.

Surveillance practitioners charged with dealing with the data will bring an analysts perspective focused on issues relating to data quality such as missing data, data entry errors, data standards, data definitions and other practical data processing issues. Communicating these issues to data providers will emphasize the importance of these issues and may result in improved data entry and data collection. Representation from government decision makers who set policy for food safety, biosecurity and disease control programs, and the personnel who deliver these programs will be needed. They will provide clear targets and priorities for information created from the data, and more effectively communicate the importance of this information to FAS participants who create the data. Since surveillance and food animal production are continuous processes, we expect new data to become available frequently, and suggest that stakeholders groups should be engaged in an ongoing process, through regular scheduled meetings. This will be an opportunity for surveillance practitioners to provide information updates to the stakeholders, and to build strong ongoing relationships between government decisions makers, program delivery personnel, surveillance practitioners, analysts, and FAS participants.

## Identifying Changes in Food Animal Systems

Human societies rely on ecosystem services for many things including water and food, and are therefore dependent on the health of ecosystems. Recent losses of species and other indications of ecosystem failure have prompted researchers to search for methods aimed at quantifying the health of ecosystems (58). Modeling approaches, such as those described in the previous section have been widely used to understand ecosystems, however sufficient data from ecosystems are often unavailable to parameterize these models, leading researchers to look for other methods. This search has focused on identifying indicators of change in ecosystems that are predictors of major or catastrophic shifts in the state of ecosystems (59–61). Researchers have theorized that as CAS change, there are accompanying changes in the characteristics of time series from the CAS, and research has focused on identifying metrics to quantify these changes (59–61). These methods have been applied to other CAS and some of them could potentially be useful for AHS.

**Figure 1:**
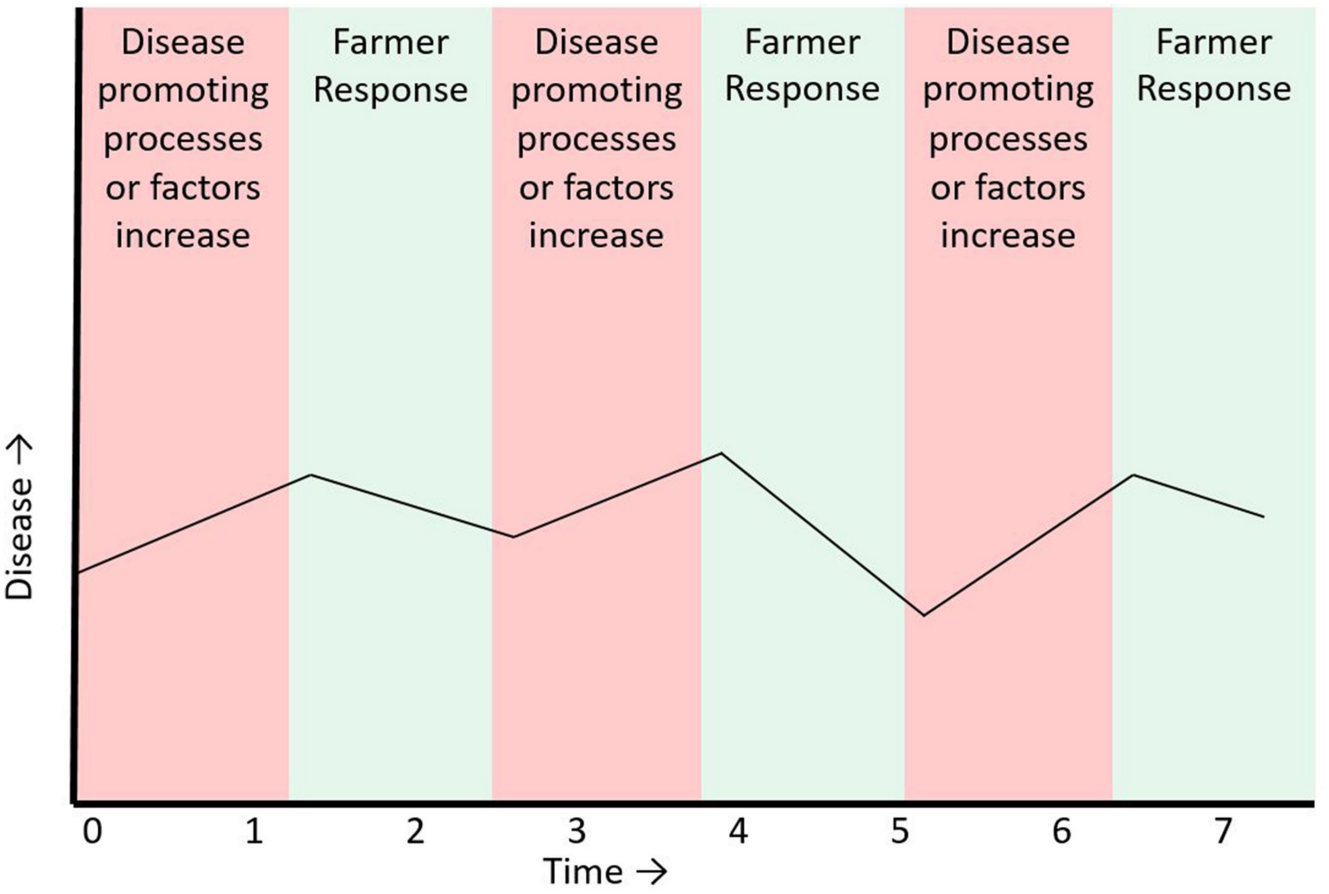
Disease in a population of farm animal as a hypothetical example of a negative feedback process with an accompanying time series of disease occurrence. During times when causal factors for disease increase (red shaded time intervals) there will be a corresponding increase in disease seen in the time series. When the farmer responds to the increase in disease (green shaded time intervals) by reducing disease causing factors, disease will goes down.

**Figure 2:**
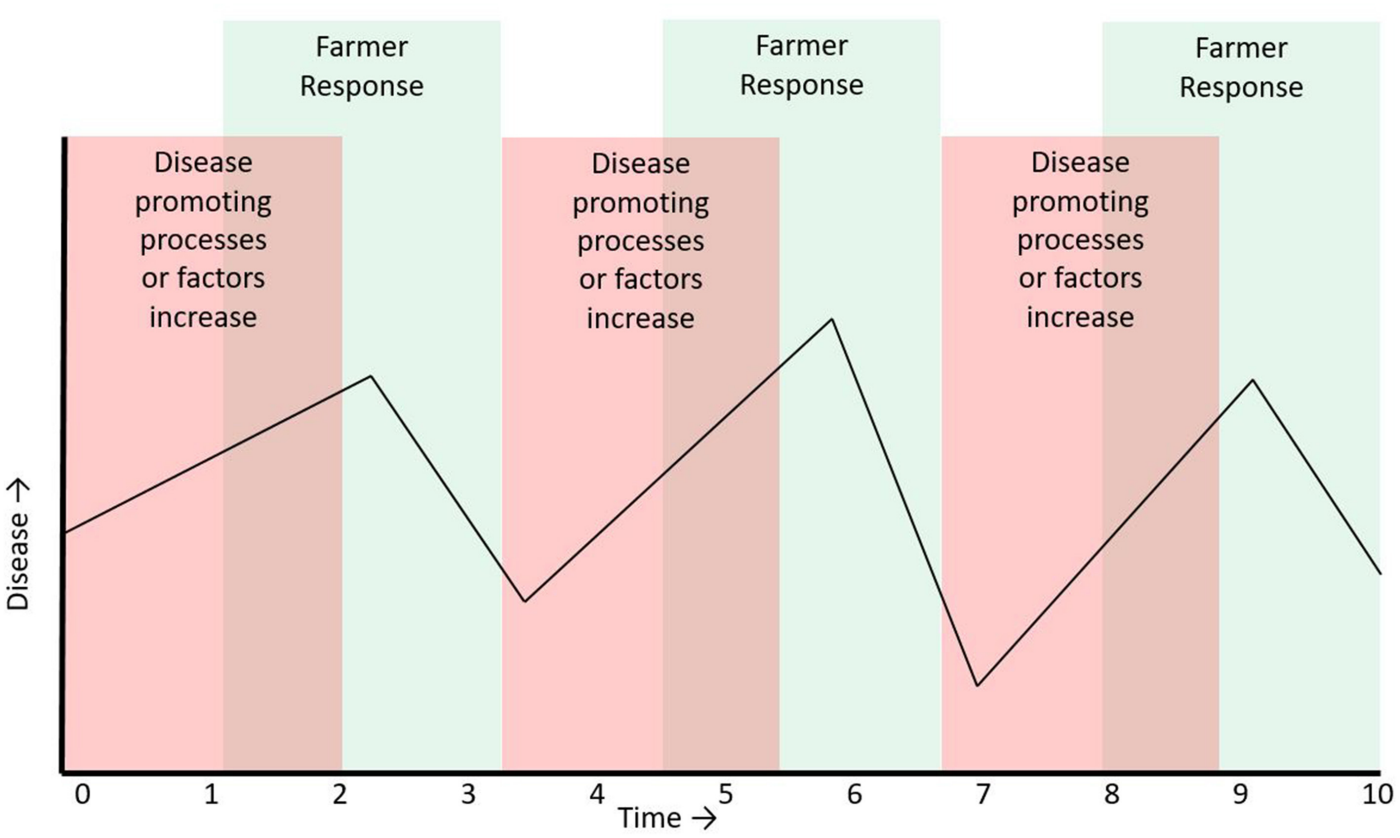
Decreasing resilience in a CAS may be due to a loss in strength of the negative feedback process. In this example the farmer's response is initially unable to reduce the effect of the causal factors for disease, as illustrated by the overlap of the green (farmer response) shaded interval with red shaded interval (time when causal factors are operating). It takes longer for the farmer's response to return disease back to an acceptable level. The resultant effect on the disease time series is an increase in variation in the time series and an increase in the time interval between peaks (observed as a slowing down of the time series).

Many of the methods developed for monitoring change in ecosystem time series are based on the idea that stability in a CAS is maintained by negative feedback processes, where movement in some quantity (or feature) away from a stable value is tied to processes that return the quantity back to a stable level. Ecosystems are constantly subjected to internal forces, or shocks, that cause movement (increases or decreases) in these quantities or features away from stable values. The stability or resilience of the ecosystem is theorized to relate to the magnitude of change that occurs in the quantity before it returns to its stable value, and the length of time that it takes to return (Figures 1, 2). If shocks are relatively constant, then larger magnitudes of change and longer periods of time to return to stable states (called critical slowing down) would be indicators of the weakening of negative feedback processes, and loss of resilience (59). The practical value of this paradigm is that it allows for measureable estimation of the stability of ecosystems. For example increased variance in ecosystem time series have been shown to be indicators of reduced ecosystem resilience (Figure 2). A number of time series metrics including: lag one autocorrelation, skewness, kurtosis, bimodality, return rate, de-trended fluctuation analysis indicator, conditional heteroscedasticity, spectral density, and others, have been proposed for monitoring changes in CAS (58, 59, 62, 63).

In a FAS, for example, disease occurrence is strictly controlled by feedback processes. At the farm level, any increase in animal disease is quickly followed by a negative responses from the farmer, such as antimicrobial or vaccine administration, or changes in production practices aimed at reducing or eliminating the disease (Figure 1). Following the ecosystem paradigm, monitoring time series of FAS disease counts could be used to estimate changes in disease production processes in a FAS (Figure 2). The characteristics of disease count time series from a FAS could be affected by both the characteristics of the shock (in this case changes in a pathogen or changes in factors that favor disease production) to the FAS, and the strength of the negative feedback loop (farmers' responses to increased disease). It follows that change in disease count time series metrics could be due to either: (1) factors that cause an increase in disease occurrence or (2) factors that weaken the feedback loop aimed at reducing disease occurrence. For example, if it was known that farmers had not changed their disease response capacity, then changes in the characteristics of disease count time series (for example increased variance or lag 1 autocorrelation) could be an indication of a change in a pathogen, the introduction of a new pathogen or changes in risk factors that cause increased disease production (including changes in farmer behavior such as reduced biosecurity, reduced preventative vaccination etc.). The benefit of monitoring these metrics over time is that they could be non-specific early indicators of reduced capacity of the FAS to deal with disease and therefore also early indicators of an impending epidemic in the animal population. Several time series metrics have been proposed and evaluated for forecasting disease emergence in simulated data for human diseases (64–67). To our knowledge these metrics have not yet been used in animal health, but there is no technical reason limiting their use in animal health.

Another way to estimate change is CAS is to assess the amount of complexity that is inherent in the system. In 2002 Costa introduced the idea of using entropy metrics applied to time series to estimate the amount of complexity in biological systems from which the time series was derived (68). The basis for using these methods is the theory that disease, pathology, and aging degrade the physiology adaptability and complexity of biological systems, and that these changes in complexity can be detected as changes in entropy metrics in time series from these biological systems (68). For example, differences in multiscale entropy of RR intervals in electrocardiogram (ECG) times series have been found between healthy people and people with congestive heart failure, and in gait time series from young and old people (69). Other applications of entropy in health include: predicting septicemia in neonates in intensive care (70), characterizing Alzheimer's disease from electroencephalogram (EEG) signals (71), characterize and differentiate EEG signals in patients with epilepsy (72), and identifying people at high risk of falling using postural time series (73).

Entropy metrics have not, to our knowledge been used to identify changes in the complexity of FAS that could be associated with changes in health status of the FAS. Even though entropy metrics have been mostly applied at the individual animal level, their utility at the FAS level should be explored. Multiple levels of a FAS from microbiomes, to individual animals, farms, corporations and the complete FAS are complex systems. They share similar properties, and entropy metrics could potentially identify changes in complexity at several levels. Furthermore, entropy metrics have been used in other CAS such as financial time series (74), and a generalized approach has been defined for detecting shifts in concepts in populations that could be adapted to time series from CAS (75). The expected benefit is that these entropy metrics may provide subtle, but early indications of changes in the disease status of the animal population in a FAS, which may be useful for early epidemic detection.

Surveillance practitioners familiar with SS should not find it difficult to adapt CSS methods in their work. Syndromic surveillance uses statistical methods to identify extreme changes in the values of time series from populations to identify epidemics in the population. The methods discussed in this section are operationally similar. They aim to identify other types of changes in time series from CAS in order to identify changes in the CAS. We encourage AHS practitioners to explore the use of these methods, as they may perform better than the current event detection algorithms and Bayesian methods used in SS.

### Expected Benefits for AHS

We propose that developing dynamic FAS models will benefit data analyses by helping to: (1) gain a better understanding of how disease is produced in the FAS in order to focus analysis on specific FAS participants and processes and to select time series that are important for disease surveillance, (2) learn how data are produced in the FAS to facilitate dealing with data quality issues and to understand how participant behavior is related to data quality, and (3) understand the validity and importance of results of data analysis, (4) provide real-time feedback to the FAS, facilitating rapid orchestrated responses and adaptation to changing environments beyond just disease threats to include economic, climatic, social and other threats.

A participatory process that engages many stakeholders will have very practical advantages for cleaning, processing and analyzing data. The data that AHS practitioners have to deal with are very complex, highly varied, often contain errors (data entry or data extraction and transmission errors), and have variable language with no standard definitions. For example, veterinary practitioner data, which represent only one of the participant groups in a FAS can contain any of the following: date, farm ID, farm location, species/sex/production type of animals, whether the consultation was a farm visit or a phone call, individual animal clinical examination data, herd examination data (morbidity, mortality, duration of illness, whether the problem is acute or chronic, other ongoing conditions in the farm population), treatment recommendations, pharmaceuticals dispensed and dispensing instructions, laboratory submissions, diagnostic laboratory data, and non-clinical data such as vaccination, nutrition, biosecurity, and other management recommendations (9, 76, 77). There are often multiple veterinary practices included in a surveillance system, and they may use different practice management software that will have different data collection and transmission formats, making it difficult or impossible to collate these data into a single database without information provided by the software providers. Surveillance analysts will not have sufficient knowledge about these data to clean, process and analyze these data. Engaging the people who produce the data will be essential for providing this knowledge. Providing FAS participants with knowledge from the analysis of FAS data may also provide incentives for participants to improve their data.

Developing a participatory FAS model that includes participants who are data providers and information users, and that can model the behavior of data providers will help to deal with some of the data quality issues present in FAS data. Issues of data quality due to farmer and veterinarian behavior, such as non-compliance and time lags, have been reported (76, 78–80). These issues bring into question the value of these data for surveillance. Rather than considering these behaviors a reason for excluding these data from analyses, they could be dealt with by modeling the effect of FAS participant behaviors on both the production of disease and the reporting of data. System Dynamics Models have been used to model FAS participant behavior for making decisions about resource allocations for surveillance and disease control (28, 32), but could also model non-compliance and reporting lags. Adopting a participatory approach could have additional benefits for both AHS and disease management control programs. These include a better understanding of FAS and the processes that result in increased disease occurrences within FAS. There is support for this expectation from other fields. Participatory approaches (called adaptive management) in dynamic policy development has been reported to make socio-ecological systems more resilient to system change such as those caused by disease (81). An example from public health is the use of participatory approaches to systems thinking to gain a better understanding of the complex dynamic processes that result in neonatal mortality in Uganda (82).

Developing dynamic FAS models that facilitate learning about the processes that result in disease production in FAS will have many benefits. The data currently generated from FAS is unprecedented. They contain continuous and simultaneously collected time series data about many potential exposures (risk or causal factors), and outcomes (productivity metrics and disease) from a large number of participants in a FAS. These data differ from the observational data that is currently used for estimating causal associations in veterinary epidemiology. Observational veterinary epidemiological studies for estimating associations between exposures and outcomes are based on data collected at one point in time (cross-sectional and case-control studies), at the beginning and end of a study (longitudinal cohort studies), or at intervals throughout the study (longitudinal cohort studies). Having access to simultaneously collected time series of an unprecedented number of exposure and outcome variables from a FAS is an opportunity to explore the dynamic relationships between multiple exposures and outcomes. Time series of exposures and outcomes will enable the identification of exposures and outcomes that are associated in time and space (i.e., that vary at the same time in the same place, or with a lag between an exposure and outcome). These new data are a resource that veterinary epidemiologists can use to develop new methods for estimating associations between exposures and outcomes and new information about causal associations in FAS. Considering the large number of exposure and outcome variables that are available in FAS, we can expect that information about new associations will be identified that will be of interest to FAS participants.

The number of time series that will have to be dealt with in FAS data is large. Analyzing or modeling large numbers of time series is relatively new to veterinary epidemiology as there are only a few methods reported and they deal only with small numbers of time series (14, 83). Looking outside the field, there are supervised and unsupervised machine learning methods that have been proposed for dealing with large numbers of variables in other fields (84, 85). Since the number of variables are large, we expect that using unsupervised analytical approaches will identify spurious (due to chance alone) or nonsensical associations. We propose using FAS participants and dynamic FAS models to identify potential exposure and outcome associations that are considered valid by FAS participants, and that are supported by the FAS model structure, flows and outputs.

Observational studies are well-known for their bias and limitations (86, 87). Engaging FAS participants to comment on the quality (incorrect or inaccurate data entry) of specific time series and the associations identified, should help to reduce bias and remove spurious associations from further analyses or generalization. Identifying valid exposure-outcome associations will allow surveillance analysts to select specific exposure and outcome time series on which to focus monitoring and event detection activities, thereby reducing the number of time series that need to be monitored for a specific disease. Having geo-located time series data to monitor both exposure and outcome variables at the same time in a surveillance system has to our knowledge not been reported. We expect these data to be produced well into the future and that data accumulated over time will create additional opportunities. For endemic or periodic diseases, associations that are identified in early years or historical data can be tested for validity in the data received in subsequent years. Those associations that are consistent over time may allow the development of prediction surveillance or pre-disease surveillance based on the monitoring of those exposure time series that have been found to be highly associated with occurrence of specific diseases. Similar prediction approaches are being tested for Food Safety applications in FAS. Tamplin reported combining microbial growth prediction models with continuous data from automated sensors (temperature, humidity, vibration) into larger predictive models that are currently being tested for predicting bacterial loads in foods produced in supply chains (88).

## Summary

Surveillance practitioners are being faced with the task of analyzing large data sets that are becoming available from food animal production systems. Current epidemiological tool boxes are not adequate for dealing with these data. We argue that food animal production systems are complex adaptive systems and that there are methods that have been developed to study CAS that could be used to deal with these new food animal production data. Applying these methods to the design and implementation of AHS should have significant benefits.

## Author Contributions

All authors contributed to the conceptual content of the manuscript. JB wrote the manuscript.

### Conflict of Interest Statement

The authors declare that the research was conducted in the absence of any commercial or financial relationships that could be construed as a potential conflict of interest. The handling Editor declared a past co-authorship with one of the authors SR.
